# Duplication of the Pituitary Gland: CT, MRI and DTI Findings and Updated Review of the Literature

**DOI:** 10.3390/brainsci12050574

**Published:** 2022-04-29

**Authors:** Bettina L. Serrallach, Ronald Rauch, Sarah K. Lyons, Thierry A. G. M. Huisman

**Affiliations:** 1 Edward B. Singleton Department of Radiology, Texas Children’s Hospital and Baylor College of Medicine, Houston, TX 77030, USA; rarauch@texaschildrens.org (R.R.); huisman@texaschildrens.org (T.A.G.M.H.); 2 Pediatric Diabetes and Endocrinology, Texas Children’s Hospital and Baylor College of Medicine, Houston, TX 77030, USA; sarah.lyons@bcm.edu

**Keywords:** duplication of the pituitary gland (DPG), DPG-plus syndrome, computed tomography (CT), magnetic resonance imaging (MRI), diffusion tensor imaging (DTI), embryology

## Abstract

Duplication of the pituitary gland (DPG) is an extremely rare malformation. DPG is associated with a wide variety of midline and central nervous system malformations (DPG-plus syndrome). We present the computed tomography (CT), magnetic resonance imaging (MRI) and diffusion tensor imaging (DTI) findings of a rare case of DPG with associated tuberomammillary fusion resulting in a hypothalamic mass-like configuration, oropharyngeal teratoma, cleft palate, hypertelorism, duplicated/broad sella, duplication/low bifurcation of the basilar artery, and craniovertebral midline anomalies. Qualitative interpretation of DTI yielded normal white matter organization of the brain. The duplication of the prechordal plate and the rostral end of the notochordal plate/notochord is thought to be the main factor leading to a duplication of the pituitary primordium and resulting in the formation of two morphologically normal glands. The time of induction of the teratogenic influence, the extent of the prechordal plate and notochordal plate/notochord abnormalities, and the faulty interactions are believed to be the reason for the wide spectrum of associated midline abnormalities.

## 1. Introduction

Duplication of the pituitary gland (DPG) is an extremely rare malformation and has been described in less than 60 patients so far [1,2,3,4,5,6,7,8,9,10,11,12,13,14,15,16,17,18,19,20,21]. DPG is associated with a broad variety of midline and central nervous system malformations and has been reported in both pediatric and adult populations [9,11,17]. The most frequently reported additional findings are hypertelorism (65%), cleft palate (61%), hypothalamic mass/(pseudo-)hamartoma (58%), duplicated/broad sella (55%), agenesis of the corpus callosum (39%), cleft/fusion of vertebral bodies (35%), cleft basisphenoid bone (32%), duplication of the optic chiasm (23%), oropharyngeal teratomas (23%), duplication of the basilar artery (19%) and aberrant circle of Willis (19%) [17]. As additional findings associated with DPG are thought to occur as part of a developmental continuum, the term DPG-plus syndrome has been proposed [11]. It was found to have a predominance in females [17]. The precise extent of associated malformations, which may be indicative of potential complications, can be depicted on multimodal neuroimaging, including CT and MRI. Many patients with DPG-syndrome do not survive the first months of life [17]. Endocrine alterations are frequent in patients with DPG-plus syndrome and these patients may present with precocious or delayed puberty [14]. The exact pathogenesis of DPG is still unknown. 

The purpose of this report is to present the imaging characteristics including an evaluation of the brainstem fiber architecture by diffusion tensor imaging (DTI) in a patient with DPG and a complex of associated midline malformations. 

## 2. Case Report

A preterm female infant (35 1/7 weeks), born to a 33-year-old, was delivered by cesarean section. The birth weight was 2018 g (18th percentile for gestational age), the length 46.5 cm (66th percentile) and the head circumference 31.5 cm (48th percentile). Pregnancy was complicated by an orofacial mass seen on prenatal ultrasound and by symptomatic polyhydramnios for which amnio-reduction was performed four weeks prior to delivery. Due to airway compromise resulting from the fetal mass, the child was intubated at the time of delivery (ex utero intrapartum treatment (EXIT) procedure). Family history was unremarkable with no history of congenital malformations or hereditary diseases.

**Figure 1:**
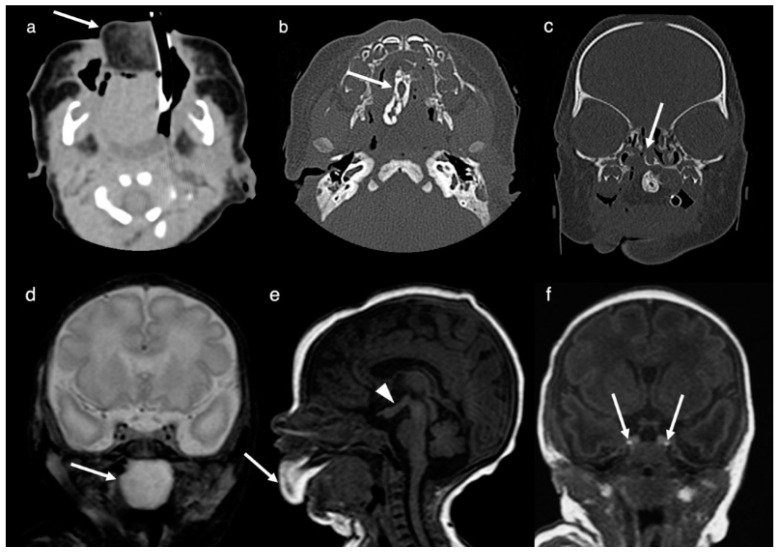
Images obtained at the second day of life. Axial CT-image (**a**), axial CT-image (bone algorithm, (**b**)), coronal CT-image (bone algorithm, (**c**)), coronal T2-weighted MR-image (**d**) and sagittal T1-weighted MR-image (**e**) show a heterogeneous mass centered in the oral cavity and protruding anteriorly through the mouth. The mature teratoma is composed out of fatty tissue anteriorly (arrows in (**a**,**e**)), a mineralized/bony component in the midline (arrow in (**b**)) and a cystic component posteriorly within the naso-/oropharynx (arrow in (**d**)). A cleft palate with corresponding defect in the hard palate can be seen in (**c**) (arrow). The tuberomammillary fusion is shown in (**e**) (arrowhead). Coronal T1-weighted MR-image (**f**) documents a complete duplication of the pituitary gland including the pituitary stalk. The bright spots of the duplicated posterior pituitary are clearly depicted (arrows in (**f**)).

Neuroimaging (CT and MRI) was performed on the second day of life. MRI and CT demonstrated a large (4.7 cm × 3.0 cm × 2.5 cm), irregular, and heterogeneous mass centered in the oral cavity and hard palate and protruding anteriorly through the mouth. The lesion was composed of fatty tissue anteriorly, heterogeneous soft tissue throughout, a mineralized/bony component in the region of the midline of the hard palate and a cystic component posteriorly within the naso-/oropharynx. Imaging features were consistent with an oropharyngeal teratoma (Figure 1). A cleft palate with a corresponding defect was present. Further, a complete duplication of the pituitary gland including the pituitary stalk, a tuberomammillary fusion resulting in a thickening of the third ventricle floor and a partial duplication of the distal basilar artery was noted. Hypertelorism was present. 

Endocrine evaluation of pituitary function was normal after birth. In her second week of life, she underwent surgical removal of the oropharyngeal mass. Tumor histology confirmed the diagnosis of a mature teratoma. At about 10 months of age, she had surgical correction of the hard and soft palate through Furlow’s palatoplasty, followed by bilateral commisuroplasty with tongue tie at three years and a frenulectomy at four years of age.

**Figure 2:**
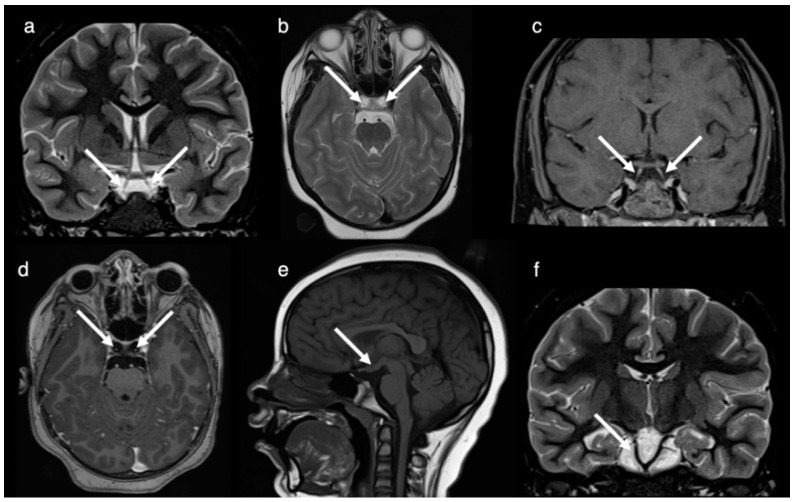
Brain MRI images obtained at 8 years of age. Coronal T2-weighted short-tau inversion recovery (STIR; (**a**)), axial T2-weighted (**b**), coronal fat saturated T1+C-weighted (**c**) and axial T1+C-weighted (**d**) MR-images demonstrate the complete duplication of the pituitary gland including the pituitary stalk (arrows in (**a**–**d**)). Sagittal T1-weighted MR-image (**e**) documents the tuberomammillary fusion (arrow). Coronal T2-weighted STIR image (**f**) shows the duplication/low bifurcation of the basilar artery (arrow) at the midpons level with the distal basilar artery supplying the superior cerebellar and posterior cerebral arteries.

**Figure 3:**
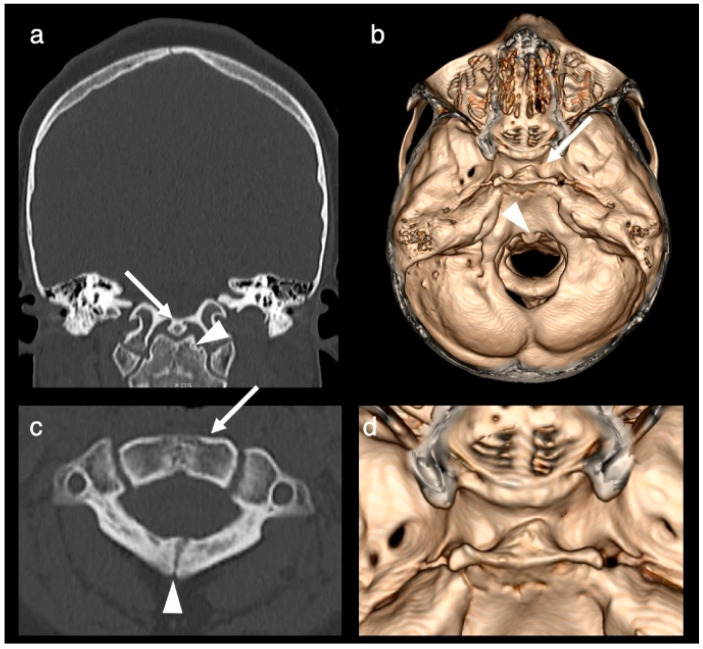
CT obtained at the age of 8 years. Coronal CT-image (bone algorithm; (**a**)), axial CT-3D reconstruction (**b**), axial CT-image (bone window, (**c**)) and axial CT-3D reconstruction (**d**). Images demonstrate a broadened/duplicated odontoid process (arrowhead in (**a**)), a bony fusion of a midline ossicle with the clivus (arrow in (**a**) and arrowhead in (**b**)), a broadened base of C2 (arrow in (**c**)), a congenital absence of the anterior arch of the atlas (**c**), an incomplete fusion of the posterior synchondrosis (arrowhead in (**c**)) and a broad and shallow sella turcica, which is divided into two fossae by a midline osseous ridge (arrowhead in (**b**) and close up in (**d**)).

The patient was seen at 8 years of age for the diagnostic work-up of headache including a follow-up MRI and CT. Imaging redemonstrated the complete duplication of the pituitary gland including the pituitary stalk, the tuberomammillary fusion with thickening of the third ventricle floor and the low bifurcation of the basilar artery at the midpons level with the distal basilar artery supplying the superior cerebellar and posterior cerebral arteries (Figure 2). A broadened base of the sphenoid with a broad and shallow sella turcica divided into two fossae by a midline osseous ridge (Figure 3) and a broadening of the nasal cavity were evident. At the level of the craniocervical junction, a congenital absence of the anterior arch of the atlas, an incomplete fusion of the posterior synchondrosis, a broadened base of C2 and odontoid process and a bony fusion of a midline ossicle (possibly the secondary ossification center/persistent ossiculum terminale) with the clivus was seen (Figure 3). Hypertelorism was confirmed. The central nervous system was otherwise normal, specifically normal corpus callosum and olfactory bulbs and tracts.

**Figure 4:**
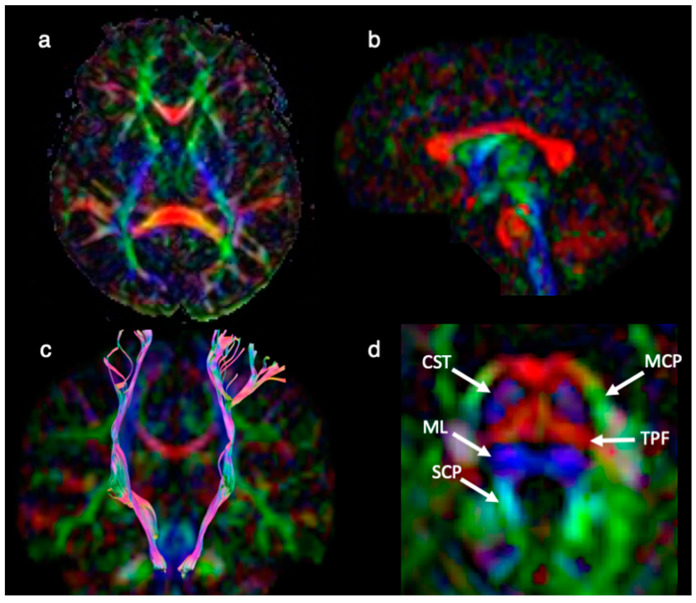
DTI obtained at 8 years of age. Axial (**a**), and sagittal (**b**) color-coded fractional anisotropy (FA) map showing qualitative normal white matter organization of the brain. Coronal fiber tractography superimposed on color-coded FA map (**c**) displays the normal course of the corticospinal tracts (CST) on both sides. Axial color-coded FA map at the level of the middle pons (close up, (**d**)). The principal diffusion direction within the areas of high degree anisotropic diffusion is color-coded in red (left to right diffusion), blue (superior-inferior diffusion) and green (anterior-posterior diffusion). At the level of the middle pons (**d**), the CST (anteriorly) and the medial lemniscus (ML) (posteriorly) can be appreciated as blue vertical structures. Between the CST and ML, the transverse pontine fibers (TPF) can be seen as a red structure. The medial cerebellar peduncle (MCP) can be detected as a green structure laterally to the brainstem. The superior cerebellar peduncle (SCP) is shown as a light blue-green structure (due to a slightly vertical orientation) medially to the MCP.

DTI, a technique to render information on the central nervous system fiber ultrastructure [22,23], revealed normal qualitative white matter organization of the brain, in particular, there was a normal configuration of the major white matter bundles within the mesencephalon and brainstem (Figure 4).

Throughout most of her childhood, she has had tall stature (>97th percentile) and body mass index in the obese range (>97th percentile). Premature adrenarche was diagnosed at age 5 years (laboratory evaluation notable for elevated dehydroepiandrosterone sulfate with prepubertal response to gonadotropin-releasing hormone stimulation test). At ages of 4 and 5 years, phalangeal bone age assessments per standard of Greulich and Pyle were performed, both times showing bone age older than chronologic age but within two 2 SD for chronologic age. An additional hormonal evaluation revealed normal thyroid function, normal insulin-like growth factor (IGF) 1 and IGF-binding protein 3, and concentrated urine osmolality ruling out diabetes insipidus. The cortisol response to the low dose cosyntropin stimulation test was normal at age 5 years. She had a repeat low dose cosyntropin stimulation test at age 7 in the assessment of fatigue; cortisol response was suboptimal concerning a new diagnosis of adrenal insufficiency. 

## 3. Discussion

DPG is an extremely rare malformation [1,2,3,4,5,6,7,8,9,10,11,12,13,14,15,16,17,18,19,20,21]. To our best knowledge, this is the first case report to apply DTI in the context of DPG-plus syndrome. Qualitative interpretation of DTI yielded normal white matter organization of the brain, in particular, a normal distribution, location and size of the major fiber tracts within the mesencephalon and brainstem.

In our patient, in addition to the DPG, a tuberomammillary fusion resulting in a hypothalamic mass-like configuration, an oropharyngeal teratoma, a cleft palate, hypertelorism, a duplicated/broad sella, a duplication/low bifurcation of the basilar artery, and craniovertebral anomalies were noted. This is in accordance with the literature, where DPG is seen as a syndrome continuum with a wide variety of associated malformations (DPG-plus syndrome) [11].

Traditional embryological studies postulated that the pituitary gland forms by a fusion of the craniopharyngeal evagination from the roof of the oropharyngeal membrane growing upwards towards the neural tube and a downward extending neuroectodermal evagination from the floor of the diencephalon [9]. There is, however, growing evidence that the pituitary gland develops as a single structure, despite the fact that it contains two independent segments (neuro- and adenohypophysis) [9,24]. It is believed that the close topographical relationship between the prechordal plate, the notochordal plate, the hypophyseal primordium and the oropharyngeal membrane during early embryogenesis gives rise to the development of the pituitary gland [9,24]. The primordium with its regulative capacity and plasticity is seen as the source of organ duplication [9,24]. Among others, Kollias and colleagues see the duplication of the prechordal plate and the rostral end of the notochordal plate/notochord as the main factor leading to a duplication of the pituitary primordium (area of the neuroectodermal adherences) and resulting in the formation of two morphologically normal glands [9,13]. The time of induction of the teratogenic influence, the extent of the prechordal plate, notochordal plate/notochord abnormalities and the faulty interactions are thought to be the reason for the wide spectrum of associated midline abnormalities [9,13]. Müller and colleagues could demonstrate that during normal development a bifurcation of the notochord occurs rostrally at stage 12 (between 4–5 postfertilization weeks) with the dorsal limb disappearing (by the latest at stage 14) and the ventral limb being the definite continuation [24]. Therefore, one could postulate that also an absent involution of the dorsal limb could lead to a pituitary gland duplication and associated malformations.

Our case showed qualitatively normal main fiber architecture throughout the brain. However, quantitative DTI changes could occur in the absence of visual changes. In addition, one could assume that qualitative abnormal DTI due to aberrant midline white matter architecture could potentially be found in extreme forms of DPG-plus syndrome, as partial duplication of the brain, third cerebral peduncle and diplomyelia have been associated with DPG [9,11]. As the notochord induces brain development, a notochord split extending further caudally could serve as an explanation [11]. 

A tuberomammillary fusion resulting in a hypothalamic mass-like appearance is found in 58% of patients with DPG [17]. It is attributed to the altered inductive role of the split rostral tip of the notochord on ependymal differentiation and cell migration and results in the interruption of lateral cell migration, which constitutes the hypothalamic nuclei [9]. Endocrine alterations, such as precocious puberty, hypogonadotropic hypogonadism with delayed sexual development, hypothyroidism, or hyperprolactinemia are frequent in DPG-plus syndrome and endocrinological evaluations should be performed to initiate appropriate management [14]. Endocrine anomalies could be a result of nuclear derangement and dysregulation of the hypothalamic-pituitary axis [14]. 

In addition, our patient showed an oropharyngeal teratoma. Oropharyngeal masses in general are commonly seen with DPG and a frequency of 55% is reported, with 23% attributed to teratomas [17]. It is hypothesized that a duplication of the floor of the embryonic mouth (stomodeum) may result in abnormal development of the mesenchyme which forms the facial structures around the stomodeum [9]. The inclusion of mesenchymal structures, as a precursor of a teratoma, is presumed to hinder a midline fusion leading to a cleft in the hard and soft palate [9]. As in our case, pregnancies are often complicated by polyhydramnios due to the inability of the fetus to swallow amniotic fluid [15]. Failure in the mentioned midline fusion generally leads to broadened facial structures and is evident as hypertelorism in 65% of cases [9,17].

A broad/duplicated sella is often associated with DPG and is reported in 55% of cases [17]. As the cartilage of the primordial sphenoid bone develops around the adenohypophyseal lobe, a duplication of the latter leads to a broadened or duplicated pituitary fossa [9].

At the level of the craniocervical junction, our patient showed an absence of the anterior arch and an incomplete fusion of the posterior synchondrosis of the atlas, a broadened base of C2, a broad/duplicated odontoid process and a bony fusion of a midline ossicle (possibly the secondary ossification center/persistent ossiculum terminale) with the clivus. Similar anomalies seen with DPG can be found in the literature [12,14]. Bony vertebral anomalies are seen in 35% of patients with DPG [17]. It is believed that the odontoid has two ossification centers on each side of the midline and that a broadened/duplicated odontoid process is a result of a lack and/or abnormal fusion [12].

A duplication/low bifurcation of the basilar artery can be found in up to 19% of patients with DPG [17]. It is postulated that the split of the rostral end of the notochord leads to a failed fusion of the embryonic longitudinal arteries into the basilar artery [15].

Multimodality imaging combining the various specific diagnostic qualities of CT and MRI is valuable to identify the exact composition and architecture of this complex malformation. CT and MRI give complimentary information. CT is exquisitely sensitive to bony abnormalities allowing for a detailed evaluation of the skull base and craniocervical anomalies. Modern MRI techniques allow for high resolution anatomical soft tissue imaging (T1- and T2-weighted MR sequences) while advanced functional MRI sequences (diffusion weighted imaging and diffusion tensor imaging, DWI/DTI) allow for the non-invasive detailed evaluation of the microstructural neuro-architecture including information about the course, size, and integrity of normal and anomalous white matter tracts. Combining the various qualities of information, these specific data will assist in making decisions about treatment options (e.g., surgery, endocrinology), and may prevent or limit future complications (e.g., respiratory complications, cerebrospinal fluid leakage after skull base surgery, cranio-cervical instability), allowing for patient and parental counseling (e.g., what to expect in the future) and finally may help to better understand the embryological and etiological nature of this malformation.

In conclusion, we report a rare case of DPG with associated midline anomalies (DPG-plus syndrome). Qualitative interpretation of DTI yielded normal white matter organization of the brain. Further research focused on advanced MR imaging techniques, such as DTI to obtain detailed non-invasive qualitative and quantitative information on the neuro-architecture of white matter tracts in DPG-plus syndrome may help to give more insight into the understanding of this rare syndrome.

## Data Availability

Data are contained within the article.
